# “Breaking through the 40% adoption ceiling: Mind the seed system gaps.” A perspective on seed systems research for development in One CGIAR

**DOI:** 10.1177/0030727021989346

**Published:** 2021-01-28

**Authors:** Margaret A McEwan, Conny JM Almekinders, Jorge JL Andrade-Piedra, Erik Delaquis, Karen A Garrett, Lava Kumar, Sarah Mayanja, Bonaventure A Omondi, Srinivasulu Rajendran, Graham Thiele

**Affiliations:** 1 54718 International Potato Center (CIP), Nairobi, Kenya; 2 CGIAR Research Program on Roots Tubers and Bananas, Lima, Peru; 3 Knowledge Technology and Innovation Chair Group, Social Sciences, 4508 Wageningen University and Research, Wageningen, the Netherlands; 4 54718 International Potato Center, Lima, Peru; 5 67609 International Center for Tropical Agriculture (CIAT), Vientiane, Lao P.D.R.; 6 Plant Pathology Department, 3463 University of Florida, Gainesville, FL, USA; 7 105528 International Institute of Tropical Agriculture (IITA), Ibadan, Nigeria; 8 International Potato Center (CIP), Kampala, Uganda; 9 Alliance of 18250 Bioversity International and CIAT, Cotonou, Benin

**Keywords:** Vegetatively propagated crops, root, tuber and banana crops, varietal adoption, farmers’ demand for seed, seed delivery profile, seed quality, seed health strategy, seed regulation

## Abstract

Seed systems research is central to achieving the United Nations Sustainable Development Goals. Improved varieties with promise for ending hunger, improving nutrition, and increasing livelihood security may be released, but how do they reach and benefit different types of farmers? Without widespread adoption the genetic gains achieved with improved crop varieties can never be actualized. Progress has been made toward demand responsive breeding, however the draft CGIAR 2030 Research and Innovation Strategy fails to recognize the complexity of seed systems and thus presents a narrow vision for the future of seed systems research. This points to the lack of evidence-based dialogue between seed systems researchers and breeders. This perspective paper presents findings from an interdisciplinary group of more than 50 CGIAR scientists who used a suite of seed systems tools to identify four knowledge gaps and associated insights from work on the seed systems for vegetatively propagated crops (VPCs), focusing on bananas (especially cooking bananas and plantains), cassava, potato, sweetpotato, and yam. We discuss the implications for thinking about and intervening in seed systems using a combined biophysical and socioeconomic perspective and how this can contribute to increased varietal adoption and benefits to farmers. The tools merit wider use, not only for the seed systems of VPCs, but for the seed of crops facing similar adoption challenges. We argue for deeper collaboration between seed systems researchers, breeders and national seed system stakeholders to address these and other knowledge gaps and generate the evidence and innovations needed to break through the 40% adoption ceiling for modern varieties, and ensure good quality seed once the new varieties have been adopted. Without this, the achievements of breeders may remain stuck in the seed delivery pipeline.

## CGIAR and the challenge of world food security

The recently drafted CGIAR 2030 Research and Innovation Strategy aims to contribute to achieving five of the UN sustainable development goals (SDGs): nutrition, poverty, equity (inclusion), environment and climate change (CGIAR, 2020). As an overarching framework for the world’s largest international agricultural research for development group, this strategy has invited vigorous commentary on past accomplishments of the CGIAR (e.g. Byerlee and Lynam, 2020; Halewood et al., 2020) and discussion of future priorities (e.g. Haddad, 2020; Lobell, 2020; Thiele and Friedman, 2020). A key contribution is improving agricultural productivity in an equitable and sustainable manner to feed the world now and in an uncertain future. While breeders have made significant genetic gains capitalizing on germplasm collections, future stresses and challenges will require increased breeding efforts and more frequent varietal turnover to cope with changing conditions. However, success in translating these genetic gains from breeding programs into improved varieties in farmers’ fields has been mixed (Walker and Alwang, 2015; Eriksson et al., 2018; Thiele et al., 2020). One of the crucial technologies to increase agricultural productivity is planting material: seeds, tubers, roots, and other propagules, referred to as *seed* in this paper. Varietal turnover varies between crops and regions. In Africa, vegetatively propagated crops (VPCs), small grains and legumes hit a 40% adoption ceiling for modern varieties, while the average age of a variety found in a farmer’s field is typically 8 years or more (Spielman and Smale, 2017; Thiele et al., 2020).

This points to a mismatch between the varietal characteristics developed by breeders and those preferred or accessible to farmers, together with shortfalls in the delivery of the seeds of these varieties to different types of farmers. Moreover, once new varieties are in the hands of farmers, maintaining the quality of their seed presents challenges, especially in VPCs which are heavily affected by seed degeneration. With such mismatches, shortfalls and challenges, the investments in current breeding and seed systems are failing to achieve optimal impact. Reaching the potential contribution to achieving the SDGs from breeding and seed investments will necessitate breaking through the 40% adoption ceiling and ensuring good quality seed once the new varieties have been adopted. Meeting this challenge will require a new configuration of international research organizations and partners which should be put at the heart of the One CGIAR. The draft 2030 Research Strategy states that “CGIAR will also accelerate the scaling out of new varieties and breeds into widespread use through innovative public–private partnerships to help develop strong seed, livestock and fishery systems that maximize farmer access” (CGIAR, 2020: 25). However, while there are many forms of public–private partnerships, this vision presents an oversimplified role of seed systems, and neglects to consider that seed systems contribute to a range of goals which may require different approaches.

Considering the challenges posed by the 40% barrier and the need for varietal turnover, CGIAR-related initiatives to upgrade breeding programs to be more responsive to demand, such as the Excellence in Breeding Platform and Crops to End Hunger, are encouraging. They show that national governments and international donors still see the value in generating improved varieties of food crops for smallholder farmers in developing countries through breeding, which historically has been the core mandate of the CGIAR system. The emphasis on “getting the traits right” by being more gender-responsive and piloting methods to collect data on farmer and consumer preferences at scale (e.g. Marimo et al., 2020; Van Etten et al., 2019; Weltzien et al., 2019) raises expectations that genetic gains achieved by the breeders result in measurable improvements in farmers’ fields. Improved yield potential, disease resistance, and drought or salinity tolerance, as well as market preferences and nutritional traits will result in varieties that are more attractive to, and beneficial for, a wider range of farmers. However, the requirement to tailor and bundle all varietal traits for diverse needs and preferences remains challenging, and additional efforts will be required to break through the 40% adoption ceiling.

## Mind the seed system gap(s)

In this perspective paper we discuss how the adoption challenge also depends on seed system research and interventions and how this can be tackled from the perspective of VPCs. These crops are important when we consider their unique contribution to food security and poverty reduction, especially in low and middle income countries and among poorer households (Wiebe et al., 2020). We lay out the current and future challenges for VPC seed systems, focusing on bananas (especially cooking bananas and plantains), cassava, potato, sweetpotato, and yam. We point out that seed systems are not simply the link between breeders and farmers to multiply and deliver seed. Instead, they are complex biophysical and socioeconomic systems which encompass and link formal and informal seed sectors. We highlight four important knowledge gaps with implications for how we think about and intervene in seed systems:

1. how variety and seed demand characteristics of different types of farmers can be captured, and how these demand characteristics relate to seed acquisition behavior and translate into varietal change;
2. how seed production and delivery pathways are conditioned by interactions between biophysical and social factors;
3. how to implement an integrated seed health strategy and minimize seed degeneration, by managing the spread of pests and diseases through seed and in the field;
4. how seed policy and regulation frameworks to manage diseases facilitate or impede availability and access to quality seed.

We argue that without better understanding of the mismatches and methodically addressing these gaps in our knowledge of seed systems, the achievements of breeders may remain stuck in the seed delivery pipeline. In so doing we highlight the importance of a wide range of interacting factors—seed quality, demand, multiplication, and delivery mechanisms—which together define the emergent characteristics and performance of a seed system. This underlines the need to address seed systems research for development (R4D) in a holistic and coherent way. These interactions are also context specific, urging a rethink of how seed system interventions relate to agri-food systems and their markets. Finally, we argue for the need to operationalize deeper collaboration between researchers and national seed system stakeholders to jointly build sustained capacity for R4D of seed systems. Collaborative efforts are needed to address these and other knowledge gaps and generate the evidence and innovations needed to break through the 40% adoption ceiling and ensure good quality seed once the new varieties have been adopted.

## Addressing seed system gaps in VPCs

Seed systems are indispensable for bringing breeding results to farmers’ fields. But they are complex, engaging multiple stakeholders, and are not simple one-way delivery channels. The old linear approach of developing and “handing over” varieties to public and/or commercial entities to multiply and distribute is generally acknowledged to have been inadequate in most contexts, but especially so in the case of VPCs, open pollinated varieties (OPVs), and neglected crops. The seed systems of VPC crops are predominantly informal, and the uptake of seed production by the private sector has generally been low. For the same reasons, the public sector has difficulty sustaining early generation seed production. This is compounded by unclear mandates for the commercialization of seed between public sector research and extension and so, there is a failure to make quality seed accessible to farmers. This is exacerbated by the chronic under investment in building national capacities to systematically diagnose seed system bottlenecks and design appropriate interventions, together with appropriate supporting policies and regulations, to promote the production and distribution of seed.

Seed systems of root, tuber, banana, and other VPCs need special attention because they are different from those of crops that are propagated through botanical seed. The low multiplication ratios, the bulky and perishable nature of the planting material, and issues with pest and disease accumulation make storage, transport, and particularly seed quality, important factors shaping these seed systems. Using vegetative propagation, farmers can reasonably reproduce the same genetic material for many seasons, making seed degeneration and varietal replacement the key drivers of seed demand (Almekinders et al., 2019b). Nevertheless, investments in the productivity of root, tuber, and banana crops, predominantly grown by the poor, have high impacts on poverty alleviation (Wiebe et al., 2020).

The work of the *Seed Systems community of practice of the CGIAR Research Program on Roots, Tubers and Bananas (RTB)* has been focusing on understanding these bottlenecks. This community comprises more than 50 scientists and practitioners from different disciplines and six organizations. It uses inter- and trans-disciplinary methods to diagnose, evaluate, and improve seed systems for banana, cassava, potato, sweetpotato, and yam. The RTB seed community has developed a toolbox comprising research, diagnostic and planning tools (Andrade-Piedra et al., 2020, https://tools4seedsystems.org/).

One tool that has been used to kick-start a systematic and comprehensive analysis of seed system bottlenecks and gaps, and subsequently monitor an intervention is the *multi-stakeholder framework* (Bentley et al., 2018, 2020). Experiences across different crop and country contexts have shown that this tool provides a basis for joint discussion among actors to identify priority areas for further research or intervention (Andersen et al., 2019; Bentley et al., 2018). The deployment of the tools and reflections on their findings across case studies allowed us to first identify and then address these four gaps in seed systems knowledge.

### Gap 1: Capturing the demand characteristics of different types of farmers

Farmers’ demand for varieties and seed defines the interface of the formal and informal seed sectors for most crops and situations and is an essential driver of seed systems. While current seed system strategies advocate demand-responsiveness and demand-orientation, we repeatedly find that farmers’ demands for quality seed of improved varieties is disappointing (e.g. Eriksson et al., 2018; Thiele, 2020). Thus, while there is increased awareness of the need for broader stakeholder involvement in breeding programs for trait prioritization for different market segments (see Excellence in Breeding Module One^1^); there is an assumption that this alone will generate the expected farmer demand for seed. We argue that this overlooks the complexities of farmer demand which also consists of preferences for types, volumes, quality, supply/delivery mechanism, and other characteristics of the seed beyond genetic identity.

Research that aims to capture preferred varietal traits and the willingness of farmers to pay for traits and seeds increasingly considers gender-related differences between farmers. For VPCs this is an especially important consideration, since in many cases these crops are part of the female domain as they are important for household food and income security. RTB studies show complex gendered differences in seed demand beyond agronomic or culinary trait related preferences. For example, Mudege et al. (2015) found in Malawi that women had less access to potato seed because they could either not afford it or depended on their husbands who do the cash purchasing in local markets. Other forms of social differentiation are also important when seeking to understand the factors affecting farmers’ seed and variety choices. Age, education, household size, and income can contribute to different preferences, as does distance to the market for products. In addition, farmers’ needs and preferences for varieties and seeds are contextual; trait-elicitation data based on surveys or visits to demonstration or trial plots probably do not reflect a farmer’s social position, the role of the crop in her livelihood strategy, market linkages and agro-ecological context (Almekinders et al., 2019a). Farmers’ real-life seed choices are balanced with trade-offs with other livelihood activities that make demands on labor, such as time, cash, and exposure to risk. But, the ways in which farmers’ decisions and seed-sourcing behavior are affected by market conditions, climate change, and other drivers remain largely understudied. Farmers may also want information about agronomic management practices such as fertilizer requirements and the need for repeated and timely weeding which have effects on varietal performance. If farmers conclude that growing the variety successfully will require additional investments, and thus loans and increased risk, they may choose another option. Similarly, in a simulated auction, farmers may readily show willingness to pay for seed. But, experiences with pre-ordering of potato seed have shown that the final proof of the pudding is in the actual purchase: when it comes to handing over the seed, many do not pay (EO Atieno, personal communication).

### Gap 2: Identifying effective seed delivery pathways

The multiplication and flow of seed along the value chain for VPC varieties that have been released or cleaned-up usually moves from formal to informal, to end up in localities and with the actors who make the seed available and accessible to farmers. But, how different seed delivery pathways^2^ link to different types of farmers has had limited research attention.

The seed delivery pathways and the intermediaries involved vary by crop, variety, and form of seed. The final seed delivery actors function as intermediaries between the formal and informal systems; they include agro-dealers, seed traders, farmers who directly receive materials from government, seed companies, or NGOs. Research on banana seed systems in Uganda shows that tissue-cultured banana plantlets travel a different route than suckers, and the former are provided by actors from which smallholder farmers and women are less likely to source their banana planting material (Kilwinger et al., 2020). A study on potato seed of improved varieties in Ethiopia indicated that the strategy of an NGO to provide better-off farmers with seed made sense, as these farmers shared seed more often than poorer farmers (Tadesse et al., 2017). Understanding the linkages between the formal and informal seed systems, and within the informal ones, is essential for identifying the most appropriate seed delivery pathways for different combinations of crops and end users. However, under many national seed regulatory frameworks informal seed systems remain *de jure* illegal.

Biofortified varieties of root, tuber, and banana crops can make direct contributions to achieving the SDGs by improving the nutrition of vulnerable groups, especially expectant and lactating women and children under the age of 5 years, for example orange-fleshed sweetpotato (Shikuku et al., 2019), and golden banana (Paul et al., 2018). However, it cannot be assumed that these groups automatically benefit from the general availability of these varieties, thus seed delivery will need to be accompanied by a series of enabling measures. A combination of nutrition education during ante-natal clinics and providing pregnant women with vouchers to stimulate demand for vitamin A rich OFSP and coordinating that with its supply via OFSP vines through the agriculture sector is one novel approach (Cole et al., 2016). This is an example of a seed delivery pathway designed to take specific consumer needs and seed sourcing behaviors into account. These and other seed delivery pathways must also consider how their attractiveness, to both seed producers and farmers, is conditioned by subsidies, access, and the costs of other inputs needed to grow, consume or market crops.

Effective seed delivery pathways depend on good quality and affordable early generation seed (EGS), however inconsistent funding has led to “stop-go” EGS production. RTB researchers have developed a Sustainable Early Generation Seed Business Analysis Tool (SEGSBAT) which calculates and plans seed production requirements for financially sustainable root, tuber, and banana early generation seed multiplication. Use of this tool has highlighted that current methods for estimating seed requirements for production planning are inadequate; that developing detailed cost structures can highlight where production costs can be reduced, e.g. through optimizing tissue culture production to screenhouse capacities; and that through the use of a revolving fund mechanism 6 out of 11 countries in sub-Saharan Africa were able to improve continuity of funding and meet at least 90% of their recurrent seed production costs from season to season (Rajendran and McEwan, 2020).

### Gap 3: Ensuring seed health and stopping the spread of disease

Seed multiplication is subject to the build-up of many pests and diseases that are carried over to the next generation, resulting in seed degeneration. This risk is especially important for vegetatively propagated crops. While seed degeneration and its impacts on yield have been widely studied for some crops such as potato, many unknowns remain. These form what we call a “seed health knowledge gap” that can be addressed by using the following tools (strategies, models, frameworks) which in turn help to design better seed interventions.

An integrated seed health strategy, effectively combining healthy seed, resistant varieties, and good on-farm management, can be used to slow seed degeneration (e.g. Thomas-Sharma et al., 2016). When seed multiplication practices are effective, higher seed quality allows profitable yields over more growing cycles, although yields still may be far below potential. A central tool for formulating integrated seed health strategies is developing pathosystem-specific seed degeneration risk-assessment models and decision support systems to define key management components, such as renewal with healthy seed, to keep yield loss below a threshold (Thomas-Sharma et al., 2016, 2017). Such models can inform economic cost-benefit analysis along the seed value chain, as influenced by the price of clean seed for farmers and threshold values to understand, for example, optimal replacement rates in sweetpotato in the Lake Zone in Tanzania (Ogero et al., 2019); and strategies to limit the spread of invasive pathogens (Andersen et al., in revision). Mapping the performance of seed health management strategies in a range of environments can identify locations where investments in extension and farmer support are most likely to be effective (Buddenhagen et al., 2020).

Increased understanding of how linked socioeconomic and biophysical networks interact to affect adoption of improved varieties, and regional crop health and productivity contributes to the design of seed systems (Garrett, 2020; Garrett et al., 2018). Impact network analysis (INA) is a scenario analysis framework which has been used to evaluate gender effects on information and seed access, and disease risk in a potato seed system in Ecuador (Buddenhagen et al., 2017); to identify risk and mitigation options based on pathogen spread by both seed trade and disease vectors in sweet potato in Uganda (Andersen et al., 2019); to identify how the spread of cassava mosaic disease (CMD) interacts with patterns of stem exchange in South East Asia (Delaquis et al., 2018); and to inform policy makers on options to monitor and reduce the spread of CMD in Southeast Asia’s commercial cassava industry (Andersen et al., in preparation).

Understanding seed degeneration is crucial for developing effective interventions in seed systems, especially for VPCs, and for ensuring optimal seed quality and seed replacement rates for farmers. These in turn provide entry points for the introduction of new varieties and trait prioritization for breeding programs.

### Gap 4: Effective policies and regulation

Finally, there is a gap in adequate policy options and recommendations for what is feasible and economically viable. This gap involves regulatory issues around variety registration, seed commercialization, farmers’ rights, regional harmonization of legislation, and combating counterfeit seed.

However, the political economy around these regulatory processes can be highly contested (Scoones and Thompson, 2011). Understanding seed regulatory frameworks and their implications for seed producers and users is the first step toward acknowledging trade-offs. Examples from the seed potato industry in Kenya have demonstrated the divergent priorities of different actors, which include increasing smallholder livelihoods, living up to international phytosanitary standards, and managing reputational risk. These dynamics may help to explain the limited effectiveness of current regulatory schemes in increasing the availability of quality seed to smallholders and containing the spread of pests and diseases (McEwan et al., 2021), and is an example of coordination breakdown among seed system actors (Bentley et al., 2018). In Vietnam, while a strict regulatory regime exists, there is weak capacity for enforcement. At the local level quality is signaled through trust and reputation, but this is not feasible for national or regional trade in potato and cassava planting material. This underlines the need for regulatory approaches that can balance more permissive regimes at the local level with stricter surveillance for cross border trade (Gatto et al., 2020). The gap between ambitious regulations and what is economically feasible in practice is a common obstruction for seed producers and buyers alike. In a Nigerian case with cassava, and yam, the regulations in place were onerous for many seed producers for compliance. Working with regulatory bodies, RTB researchers launched a web-based app to simplify compliance to national seed certification procedures, which also provide an interface linking certified seed producers, regulators and buyers (www.seedtracker.org). Gaps in policy adherence and traceability in other contexts remain important limiting factors in developing EGS delivery pathways.

## The linkages between these gaps and the need for a coherent and holistic research and intervention agenda

These four seed system knowledge gaps are interconnected. Characterizing the interactions between different aspects can lead to understanding the potential viability of a range of approaches to deliver high quality seed of improved varieties to different types of farmers at sources that are attractive, convenient and accessible to them, as well as the identification of promising entry points for public and private sector engagement. For root, tuber, and banana crops this work has only just started, but is shaping a coherent effort that unifies integrated seed health models, inclusive seed business strategies, realistic approaches to understand and respond to farmers’ demand based on trait preferences and seed acquisition behavior, and policies and regulations adapted to local realities. Achieving this requires active, ongoing support to fund, design, implement, and evaluate evidence-based interventions.

The work of the RTB group illustrates the uniqueness of the seed systems of root, tuber, banana, and other VPC crops, and demands the development of alternate research agendas and methods. Increasing learning exchanges with the diverse experiences from peers in parallel crop breeding and seed programs is particularly timely. The seed systems of legumes, small grain cereal crops, and maize also face challenges in achieving the desired turnover of improved varieties, development of viable private sector engagement, and engaging policy makers to strengthen the enabling environment; challenges both similar and different to those in VPCs (Ojiewo et al., 2020; Rutsaert and Donovan, 2020). Teaming up with seed system researchers of other crops and institutions is a logical way for the One CGIAR community to further develop tools and approaches. Broader sharing of experiences across crops would enrich the tools or methods and make them more widely applicable, and additional tools, such as seed delivery profiles to design appropriate seed delivery pathways, may prove useful in systematically researching and developing more robust seed systems in a wide range of agri-food systems.

## Impact through collaboration: A call for stakeholder engagement around the gaps

Seed systems research should not be relegated to a support service function. We have highlighted four knowledge gaps and some of the implications for intervening in seed systems. To engage national stakeholders fully in tackling these and other emerging gaps, it is necessary to strengthen evidence-based dialogue with breeding programs to ensure that these challenges are sufficiently visible and to mobilize the expertise and funding to develop more intentional delivery mechanisms for different species, varieties, and seeds. Specifically, we propose co-development of seed delivery profiles with breeders and seed system scientists to provide guidance to design effective seed delivery pathways. Second, the RTB seed systems community of practice has a validated suite of core seed system research tools to address these knowledge gaps and are now investing in a process of socializing their use and the insights generated. We are redoubling efforts to expand collaboration among seed system actors, along with specialists in CGIAR centers and international initiatives, such as the Excellence in Breeding and Gender Platforms, as well as CGIAR scaling experts. Third, we are building on existing partnerships with the National Agricultural Research and Extension Systems and national seed practitioners who are primarily responsible for the seed multiplication and commercialization of VPCs and OPVs. In seeking to leverage the expertise and insights available to strengthen and sustain national capacities for delivering seed, we are co-creating a functional space for collaboration with development partners, seed systems researchers, and end users. Readers of this article are likely to find themselves in one or more of these categories, and we invite broad participation and feedback in the co-creation process.

The formulation of One CGIAR is an opportunity to delineate a new strategy for collegial breeding and seed system work in relation to the many challenges raised by the interplay between the socioeconomic and biological aspects of seed system R4D. It is important to ensure that seed systems R4D and interventions are recognized as a cross-cutting concern which requires integration across the three proposed One CGIAR action areas: systems transformation, resilient agri-food systems, and genetic innovation, so avoiding another research silo. Investments in new varieties and better seeds will only have significant impacts if they break through the 40% ceiling and lead to wide adoption of nutritious, productive and resilient varieties in farmers’ fields. Substantial progress has been made during the past 6–8 years on understanding seed systems and developing appropriate and necessary tools for seed systems interventions. This positions programs to transition smoothly into One CGIAR action areas and build effectively on these achievements to break through the 40% ceiling. The vision of teaming up and engaging in interdisciplinary action-research oriented collaboration around seed systems and seed delivery can only become a reality if the importance of seed systems and their challenges to achieving institutional and global development goals is framed as a primary and pressing pathway to impact. In this article we have outlined four key gaps which can serve as priority areas of collaboration.

## Notes

1. https://excellenceinbreeding.org/module1.

2. We define seed delivery pathways as the crop and context (agro-ecological, socioeconomic and biophysical) specific routes through which seeds are delivered to farmers. This route reflects the context and characteristics of different actors (starting with breeders and ending with the farmer him/herself) how this influences their institutional arrangements (agreements and transactions) and accompanying enabling measures needed. Within a pathway, those actors engaged in seed production and distribution enterprises may have a business model describing the value proposition for their enterprise or product and how it will generate financial or other types of value.
